# Real-World Evaluation of Dupilumab in the Long-Term Management of Eosinophilic Chronic Rhinosinusitis with Nasal Polyps: A Focus on IL-4 and IL-13 Receptor Blockade

**DOI:** 10.3390/medicina60121996

**Published:** 2024-12-03

**Authors:** Nicola Lombardo, Aurelio D’Ecclesia, Emanuela Chiarella, Corrado Pelaia, Debbie Riccelli, Annamaria Ruzza, Nadia Lobello, Giovanna Lucia Piazzetta

**Affiliations:** 1 Otolaryngology Head and Neck Surgery, Department Medical and Surgical Sciences, University “Magna Graecia” of Catanzaro, 88100 Catanzaro, Italy; debbie.riccelli001@studenti.unicz.it (D.R.); nadialobello@gmail.com (N.L.); giovannapiazzetta@hotmail.it (G.L.P.); 2 Department of Maxillofacial Surgery and Otolaryngology, IRCCS Casa Sollievo della Sofferenza, 71013 Foggia, Italy; aureliodecclesia73@gmail.com (A.D.); ruzza_annamaria@libero.it (A.R.); 3 Laboratory of Molecular Haematopoiesis and Stem Cell Biology, Department of Experimental and Clinical Medicine, University “Magna Graecia” of Catanzaro, 88100 Catanzaro, Italy; emanuelachiarella@unicz.it; 4 Department of Health Sciences, University “Magna Graecia” of Catanzaro, 88100 Catanzaro, Italy; pelaia.corrado@gmail.com

**Keywords:** chronic rhinosinusitis, nasal polyps, dupilumab, type 2 inflammation, non-type 2 inflammation

## Abstract

*Background and Objectives*: Chronic rhinosinusitis (CRS) is a complex inflammatory condition of the nasal passages that severely impairs quality of life. Type 2 CRS is characterized by eosinophilic inflammation, driven by cytokines like IL-4, IL-5, and IL-13. These cytokines are key to CRS pathogenesis and contribute to a heavy disease burden, especially with comorbidities. This study assessed dupilumab, a monoclonal antibody targeting IL-4 and IL-13 signaling, to evaluate its efficacy in reducing the disease burden in patients with CRS with nasal polyps (CRSwNP). *Materials and Methods*: The patients received subcutaneous dupilumab for 42 weeks. The outcomes included Nasal Polyp Score (NPS); Sino-Nasal Outcome Test (SNOT-22), Numeric Rating Scale (NRS), and Visual Analog Scale (VAS) scores; total IgE; and olfactory function. *Results*: Significant improvements were observed across the NPS and SNOT-22, NRS, and VAS scores after 42 weeks. Their total IgE levels were reduced, though a transient increase in peripheral eosinophilia appeared at 16 weeks. The patients also reported substantial improvements in olfactory function and high satisfaction with the treatment, supporting dupilumab’s potential in reducing both symptom severity and inflammation in CRSwNP. *Conclusions*: These results indicate that dupilumab may be an effective treatment for CRSwNP, offering significant symptom relief, improved olfactory function, and enhanced quality of life. High satisfaction levels suggest that dupilumab may provide therapeutic advantages over the conventional CRS treatments, though further studies are warranted to confirm its long-term benefits.

## 1. Introduction

Chronic rhinosinusitis (CRS) is a multifaceted inflammatory condition that affects both the nasal mucosa and paranasal sinuses.

It presents as two main phenotypes: chronic rhinosinusitis without nasal polyps (CRSsNP) and chronic rhinosinusitis with nasal polyps (CRSwNP). CRSwNP, often associated with more severe symptoms and comorbid conditions like asthma, can be further classified based on the inflammatory pathways—most notably type 2 inflammation, which is characterized by eosinophilic activity and elevated levels of cytokines such as IL-4, IL-5, and IL-13. This type of inflammation plays a significant role in refractory CRS cases, where the traditional treatments often fail to provide long-term relief [1,2,3].

Management of CRS has historically relied on corticosteroids and surgery, but these interventions often fall short, particularly in patients with severe or recurrent CRSwNP. In recent years, a better understanding of the immunological mechanisms driving CRS has led to the development of biologic therapies.

Recently, a population of mesenchymal stem cells (MSCs) was also identified in nasal polyps, which may be implicated in the inflammation process considering their immunomodulatory properties. However, the MSCs’ contribution to the microenvironment of nasal polyps still needs to be clarified [4,5,6,7]. The two types of inflammation are often age-related: in the elderly, there is less eosinophilic infiltration, which is known to increase the risk of recurrence [8,9].

Similarly, among young patients, there is a significantly higher prevalence of allergies. These data suggest that when CRS appears in the elderly, the pathogenesis is different and less linked to allergy and eosinophilic infiltration but more to the formation of nasal polyps [10].

Biologics offer a more targeted approach by blocking specific cytokines and immune pathways involved in type 2 inflammation. These therapies have transformed the treatment landscape for patients with severe CRSwNP who are unresponsive to the conventional treatments. Biologics such as omalizumab (anti-IgE), mepolizumab and reslizumab (anti-IL-5), and dupilumab (anti-IL-4/IL-13) have shown significant efficacy in reducing polyp size, improving symptoms, and reducing the need for corticosteroids or surgery. Dupilumab in particular has become the first monoclonal antibody approved for treating CRSwNP, demonstrating strong efficacy in clinical trials. These biologic therapies not only address the underlying inflammation but also improve the quality of life of patients, reducing the frequency of symptom recurrence and the need for invasive procedures [11,12,13,14].

In this study, we present findings from administering dupilumab to a cohort of 40 CRSwNP patients, focusing on its impact on key clinical parameters such as symptom scores, polyp size, and biomarkers like total IgE and peripheral eosinophilia.

## 2. Materials and Methods

### 2.1. Study Design

This study was a single-arm, pre-post-interventional study conducted across multiple centers. It evaluated the effects of subcutaneous dupilumab administration over 42 weeks in patients with chronic rhinosinusitis with nasal polyps (CRSwNP). No randomization or control group was included in this design. Clinical outcomes, such as the Nasal Polyp Score (NPS) and SNOT-22, were measured at baseline and after 42 weeks of treatment to assess the intervention’s effectiveness.

This study was conducted at multiple centers, following ethical guidelines as stipulated in the Declaration of Helsinki. The study protocol received approval from the institutional review boards of each participating site, and all of the participants provided written informed consent prior to enrollment.

### 2.2. The Nasal Polyp Score (NPS)

To assess the Nasal Polyp Score (NPS), nasal endoscopy was performed using an OLYMPUS ENF TYPE-GP fiberscope. This procedure was conducted across multiple centers, including the ENT Department of the University Hospital “Mater Domini” in Catanzaro and the ENT-Maxillofacial Surgery Department of IRCSS Casa Sollievo della Sofferenza in San Giovanni Rotondo. This multi-center approach was specifically designed to minimize selection and characterization bias among patients. The NPS is calculated based on polyp size, with scores ranging from 0 to 4 assigned to each nasal cavity. The total NPS reflects the severity of the condition, with higher scores indicating more severe nasal obstruction and polyp burden.

### 2.3. The Sino-Nasal Outcome Test (SNOT-22)

The Sino-Nasal Outcome Test (SNOT-22), developed at Washington University in St. Louis, MO, USA, was utilized to evaluate the quality of life (QoL) of patients suffering from CRSwNP. This questionnaire consists of 22 items, each rated on a scale from 0 (no symptom) to 5 (the worst possible symptom). The cumulative score can range from 0 to 110, with higher scores signifying a greater impact on quality of life due to nasal and sinus symptoms [15].

### 2.4. The Numeric Rating Scale (NRS)

The Numeric Rating Scale (NRS) was utilized to promptly gauge the patients’ subjective perception of the disease. This metric is graded on a scale ranging from 0 (“no problem”) to 10 (“worst possible perception”). Scores are classified into three categories: 0–3 = mild symptoms; 4–7 = moderate symptoms; and 8–10 = severe symptoms [16].

### 2.5. The Lund–Mackay CT Score

An initial assessment of the facial mass was carried out utilizing Computed Tomography (CT), with the Lund–Mackay CT score utilized for evaluation. This scoring system evaluates the opacity of each paranasal sinus, assigning a score between 0 (indicating no opacity) and 2 (indicating complete opacity). The total Lund–Mackay score ranges from 0 to 24, with higher scores indicative of a more significant sino-nasal disease burden. The cumulative score spans from 0 to 24, with higher scores indicating more pronounced opacity [17].

### 2.6. The Visual Analog Scale (VAS): Assessment of Sense of Smell

Sense of smell in the patients with chronic rhinosinusitis with nasal polyps (CRSwNP) was evaluated using the Visual Analog Scale (VAS) for olfactory function.

The VAS is a widely used, validated tool that allows patients to subjectively rate their ability to smell on a scale of 0 to 100, with 0 indicating complete anosmia (no sense of smell) and 100 indicating normal olfactory function.

This scale can take various formats, but the most common version consists of a 10 cm horizontal line with “no pain” labeled at one end and “worst pain ever” at the other. Patients indicate the intensity of their pain by placing a mark on the VAS line. The severity of the pain is directly proportional to the distance, in centimeters, from the “no pain” end of the VAS line to the patient’s mark [18].

### 2.7. Safety Assessments

Adverse events were monitored throughout this study’s duration. Safety assessments included tracking injection site reactions, allergic responses, and any unexpected health issues. The participants were instructed to report any adverse effects immediately, and serious adverse events were reported to the institutional review boards in accordance with the regulatory requirements.

### 2.8. Ethical Considerations

This study was conducted in adherence with the Good Clinical Practice guidelines. Ethical approval was obtained from the relevant ethics committees before the initiation of this study, and informed consent was secured from all of the participants.

## 3. Results

### 3.1. Clinical Sample, Enrollment Criteria, and Treatment with Dupilumab

**Figure 1:**
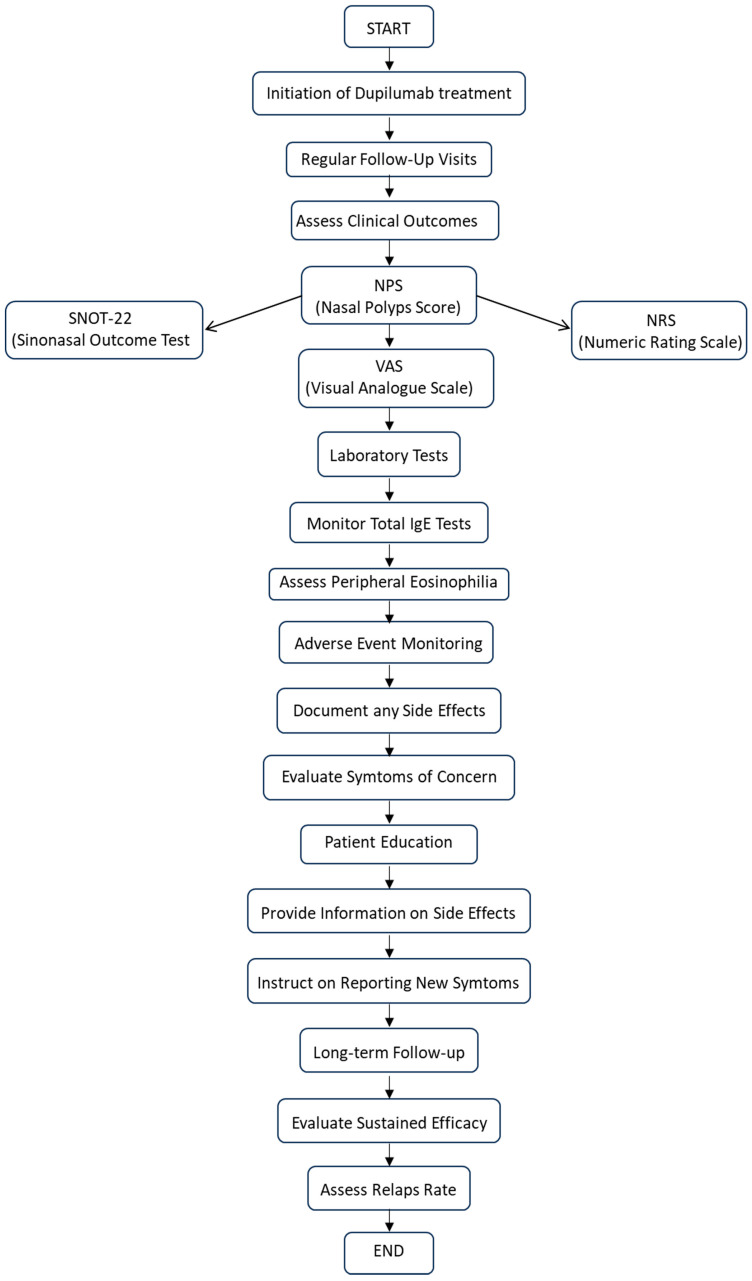
Flow chart summarizing the follow-up process for patients receiving dupilumab treatment. It outlines the key steps, including the initiation of treatment, assessments during regular follow-up visits, and the monitoring of clinical outcomes, laboratory tests, and patient education.

The clinical cohort consisted of 40 patients, of whom 38 had completed treatment for a minimum of 6 months. However, one patient discontinued treatment after the second drug administration due to psycho-emotional issues, and another was excluded due to hypereosinophilia, necessitating a switch to mepolizumab. The clinical outcomes were evaluated in this sample, comprising 31 males and 9 females aged between 31 and 79 years old, all diagnosed with chronic rhinosinusitis with nasal polyposis. A total of 45% percent of the patients had a clinical diagnosis of asthma, with 21% classified as moderate and 79% as mild. Forty percent of patients suffered from inhalant allergies, while seven had NSAID-ERD (nonsteroidal-anti-inflammatory-drug-exacerbated respiratory disease). Patients received treatment with Dupixent (dupilumab), with a 300 mg solution for subcutaneous injection administered every 2 weeks. Each single-use pre-filled pen contained 300 mg of dupilumab in 2 mL of solution (150 mg/mL). This treatment was complemented by intranasal topical corticosteroid therapy using mometasone furoate nasal spray, administered twice daily for a total of 200 mg/day. To be eligible for this study, patients had to be at least 18 years old, diagnosed with chronic rhinosinusitis with severe nasal polyposis, and have a total score on the endoscopic nasal polyposis score (NPS) ≥ 5, along with a SNOT-22 score ≥ 50. Previous treatments had to have failed due to lack of efficacy, discontinuation of systemic corticosteroid therapy, and/or a lack of response or relapse following surgery. Seventy-eight percent of the patients examined had undergone at least one Functional Endoscopic Sinus Surgery (FESS). The patient assessment included the Nasal Polyp Score (NPS), the Lund–Mackay CT score, peripheral eosinophilia, total IgE, and the impact on quality of life (QoL) assessed through the Sino-Nasal Outcome Test questionnaire (SNOT-22) from Washington University in St. Louis, MO, USA. The overall impact of the disease was evaluated using the Numerical Rating Scale (NRS). The standard guidelines on the patient recruitment flow and the different types of biases are presented in the following flow chart (Figure 1).

### 3.2. Examining the Distribution of CRSwNP Based on Age and Gender

Our study enrolled 40 patients diagnosed with CRSwNP to analyze the clinical efficacy of dupilumab. The sample comprised 45% female and 55% male patients (Table 1). The mean age of the enrolled patients was 57.4 years for men and 56.7 years for women (Table 1). The median age of the entire group of patients analyzed was 57.1. The demographic data for the CRSwNP patients enrolled in this study are detailed in Table 1. Among the enrolled CRSwNP patients, 40% were asthmatic (Table 1), with 21% experiencing moderate asthma and 79% experiencing mild asthma. Additionally, seven patients had NSAID-ERD (nonsteroidal-anti-inflammatory-drug-exacerbated respiratory disease), and 40% of patients experienced inhalant allergies (Table 1).

### 3.3. Dupilumab Treatment Led to Improvements in SNOT-22, NPS, NRS, and VAS Parameters in Patients with CRSwNP

**Figure 2:**
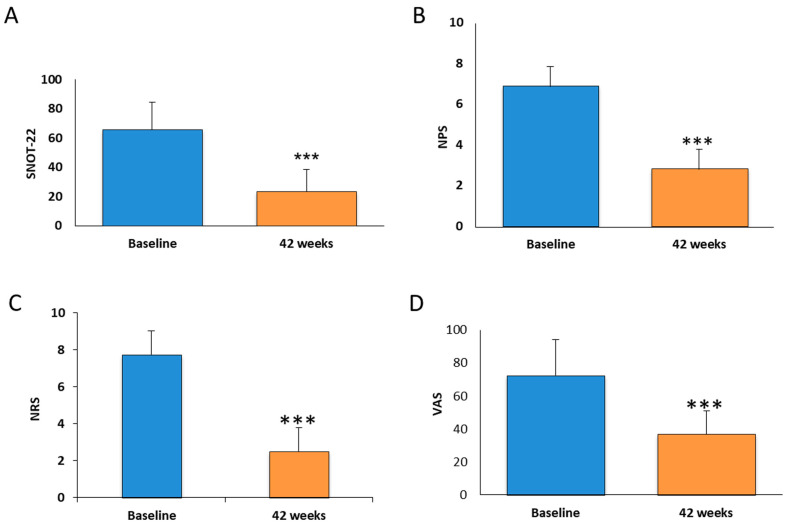
These figures depict the average values in (**A**) SNOT-22, (**B**) NPS, (**C**) NRS, and (**D**) VAS measured in 40 patients with CRSwNP undergoing dupilumab treatment for 42 weeks. These values were compared to the baseline averages. Data are shown as means ± SD from 40 patients (*** *p* < 0.0001).

The patients underwent a 42-week treatment course with dupilumab, during which their SNOT-22, NPS, NRS, and VAS parameters were evaluated, with their total IgE levels assessed at 16-week intervals. The initial parameter assessed was the score in the Sino-Nasal Outcome Test 22 (SNOT-22), a measure impacting surgical outcomes in chronic rhinosinusitis (CRS). Following 42 weeks of dupilumab treatment, their SNOT-22 values decreased by approximately 4.5 times. Specifically, the mean SNOT-22 score at baseline was 53.64 ± 22.39, which reduced to 11.85 ± 8.73 after 42 weeks (Figure 2A). Subsequent evaluation involved the Nasal Polyp Score (NPS) determined via nasal endoscopy, reflecting the extent of nasal polyp involvement. This parameter significantly improved after 42 weeks of dupilumab treatment, with the mean NPS decreasing from approximately 6.9 ± 1 at baseline to 2.8 ± 1 after 42 weeks, an approximately 2.4-fold reduction (Figure 2B). The patients’ perceived symptom severity, as measured by the Numeric Rating Scale (NRS), also improved by approximately threefold after 42 weeks of dupilumab treatment, with their scores decreasing from 7.73 ± 1.2 at baseline to 2.5 ± 1 at 42 weeks (Figure 2C). Finally, the Visual Analog Scale (VAS), used to assess pain intensity, showed significant improvement after 42 weeks of dupilumab treatment, with the baseline scores averaging 72.3 ± 22 and reducing to 36.75 ± 14 after 42 weeks (Figure 2D).

### 3.4. Assessment of Total IgE Levels in CRSwNP Patients Treated with Dupilumab for 42 Weeks

**Figure 3:**
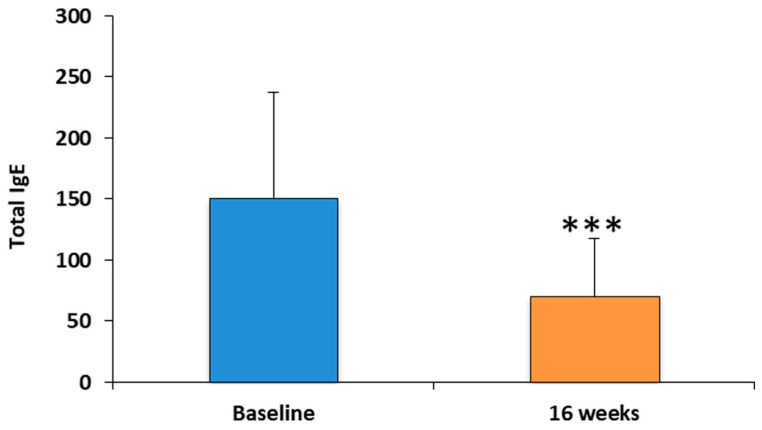
The histogram illustrates the mean values of total IgE assessed in 40 patients with CRSwNP treated with 300 m of dupilumab g for 24 weeks. The data were compared to the baseline mean values and are presented as means ± SD from 40 patients (*** *p* < 0.0001).

Total IgE levels were evaluated after 42 weeks of treatment with dupilumab. Notably, there was a significant increase of approximately 2.1 times in the patients undergoing treatment compared to baseline. Specifically, the total blood IgE data decreased from a baseline value of 150.74 kU/L to 70.15 kU/L at 24 weeks (*p* < 0.0001), as shown in Figure 3.

### 3.5. Assessment of Peripheral Eosinophilia in CRSwNP Patients Treated with Dupilumab for 16 Weeks

**Figure 4:**
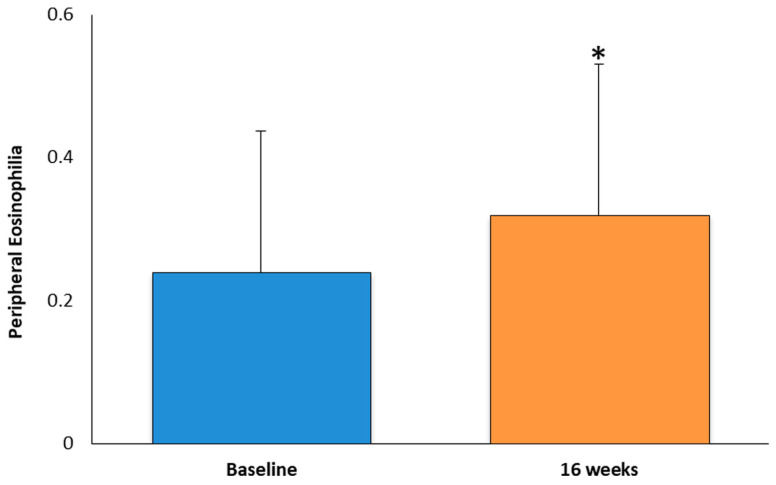
The data were compared to the baseline mean values and are presented as means SD from 40 patients (* *p* < 0.05).

Peripheral eosinophilia levels were examined after 16 weeks of treatment. Notably, there was a significant increase of approximately 1.3 times in the patients undergoing treatment compared to baseline. The mean value of peripheral eosinophils was 0.24 ± 0.21 cells/μL at baseline and 0.32 ± 0.21 cells/μL at 16 weeks (Figure 4).

### 3.6. Statistical Analysis

A statistical analysis was performed using Microsoft Excel (San Diego, CA, USA), and Matplotlib. All of the data are presented as means ± SD (standard deviation). The normality of the data distribution was assessed using the Anderson–Darling and Kolmogorov–Smirnov tests. Student’s *t*-test and the Mann–Whitney U test were utilized to compare variables as appropriate. A *p*-value < 0.05 was considered statistically significant. The average age of the patients was 56.72 ± 12.68 years old. At the outset of the treatment, the patients had a mean Lund–Mackay CT Score of 23.64 ± 1.32. Please note that verification of this information is pending as we await additional data.

## 4. Discussion

Our study demonstrated that dupilumab, a monoclonal antibody targeting IL-4 and IL-13 signaling, was highly effective in improving the clinical outcomes of patients with chronic rhinosinusitis with nasal polyps (CRSwNP). Specifically, significant improvements were observed in all of the parameters assessed, including the Sino-Nasal Outcome Test (SNOT-22), the Nasal Polyp Score (NPS), the Numeric Rating Scale (NRS), the Visual Analog Scale (VAS), total IgE levels, and peripheral eosinophilia after 42 weeks of treatment.

These findings indicate that dupilumab effectively addresses the underlying type 2 inflammatory pathways characteristic of CRSwNP. Type 2 inflammation, driven by cytokines such as IL-4, IL-5, and IL-13, is a hallmark of CRSwNP. These cytokines promote eosinophil recruitment and activation, leading to tissue damage, mucus hypersecretion, and nasal polyp formation. Through blocking IL-13 signaling, dupilumab disrupts this cycle of inflammation, reducing nasal polyps and improving mucosal function [19,20,21].

Studies have demonstrated that while dupilumab effectively reduces the symptoms of conditions associated with type 2 inflammation, such as asthma and chronic rhinosinusitis with nasal polyps (CRSwNP), its impact on IgE levels is more nuanced. Specifically, dupilumab has been shown to lead to a reduction in IgE levels in some patients, but this effect is not universal. It is essential to clarify that the clinical benefits observed in our study were not solely dependent on changes in IgE levels; rather, they reflect a more comprehensive modulation of the immune response. Research indicates that while dupilumab can inhibit IgE production by blocking IL-4 and IL-13 signaling, it does not eliminate IgE from the circulation entirely.

For instance, in a study by Hoshino et al., the authors noted that although dupilumab treatment resulted in significant clinical improvements, the overall total IgE levels varied among patients, with some experiencing no significant change in their IgE levels despite symptom relief [22]. Similarly, Castro et al. (2018) found that dupilumab reduced exacerbations and improved asthma control in patients, but their total IgE levels did not correlate directly with the clinical outcomes [23]. These findings suggest that the clinical benefits of dupilumab are not solely dependent on changes in total IgE levels.

By addressing the underlying type 2 inflammatory pathways rather than directly lowering IgE levels, dupilumab offers a unique therapeutic strategy for managing conditions characterized by eosinophilic inflammation and IgE-mediated responses.

The observed reduction in the total IgE levels in our study is consistent with dupilumab’s effect on B cells, inhibiting the switch to IgE production. IL-4 and IL-13 are critical cytokines for inducing IgE class switching, and dupilumab’s ability to reduce IgE levels in patients with CRSwNP has been noted in prior studies. These findings elucidate how the modulation of IgE may correlate with the overall reduction in allergic symptoms, particularly in patients with concomitant allergies, further supporting the therapeutic role of dupilumab in addressing type 2 inflammation beyond asthma and atopic dermatitis [24,25,26,27,28].

Our study showed a transient increase in peripheral eosinophilia after 16 weeks of treatment, a phenomenon also observed in previous clinical trials. While the increase in eosinophil counts may initially raise concern, it is important to note that dupilumab’s mechanism involves inhibiting eosinophil migration into the tissue rather than directly reducing blood eosinophil counts. We acknowledge that this transient increase may be a counterintuitive aspect of dupilumab’s efficacy and that it merits further exploration in future studies. This explains the observed peripheral eosinophilia, as circulating eosinophils are no longer being recruited into the inflamed nasal tissues. Previous studies have demonstrated that despite transient changes, eosinophil outcomes continue to improve, and eosinophil levels eventually return to baseline. Furthermore, eosinophilia-related adverse events were rare and did not compromise the treatment, a finding consistent with the literature [29].

Our results align with those from the pivotal SINUS-24 trials, where dupilumab significantly reduced polyp size, improved symptoms, and enhanced patients’ quality of life. However, our study showed a slightly greater reduction in the NPS compared to these trials [30]. One possible explanation may be differences in the baseline characteristics of the patient population, as our cohort included a higher percentage of patients with comorbid asthma, a condition strongly associated with more severe type 2 inflammation. This highlights the need for a stratified approach in future studies to understand the varying impacts of dupilumab among different patient subgroups better. The pronounced effect of dupilumab in patients with both CRSwNP and asthma has been highlighted in previous studies, improving not only nasal symptoms but also respiratory function. The marked improvement in quality of life, as indicated by reductions in SNOT-22 and VAS scores, is a critical outcome for CRSwNP patients. These improvements are particularly significant given the high disease burden associated with CRSwNP, which often leads to impaired sleep, loss of smell, and reduced daily functioning [31,32,33].

Dupilumab’s ability to restore olfactory function, as seen in our study and corroborated by others, addresses one of its most challenging symptoms [25,34,35].

As dupilumab continues to demonstrate efficacy in reducing nasal polyps and improving patient outcomes, the treatment landscape for CRSwNP is becoming more defined. However, several challenges remain. Relapse of CRS after discontinuing biologic therapy is a critical issue [36].

Biologics primarily target inflammatory pathways to reduce symptoms and prevent polyp recurrence, but they do not cure the underlying disease [37]. Therefore, the discontinuation of treatment may lead to the reactivation of inflammatory processes and a recurrence of symptoms, as seen in some patients after biologic withdrawal [38,39].

Another key consideration is the economic impact of biologic therapies. Dupilumab’s high cost may limit its accessibility, especially in healthcare systems with constrained budgets. To enhance the clinical applicability of dupilumab, identifying biomarkers that can predict which patients will benefit most from dupilumab could optimize its use and make it more cost-effective [40,41,42,43,44].

Future research should focus on defining the patient selection criteria, possibly through the development of predictive biomarkers for the treatment response. Additionally, a more detailed methodology in future studies will be critical for establishing the reproducibility and robustness of our findings. Long-term studies should assess the sustained efficacy and safety of dupilumab, particularly regarding eosinophilia and other potential immune-related effects.

## 5. Conclusions

Our study provides preliminary evidence that dupilumab may offer therapeutic benefits in patients with CRSwNP, showing significant improvements in symptom severity, nasal polyp size, and quality of life measures. While these findings suggest the potential effectiveness of dupilumab in targeting type 2 inflammation in CRSwNP, this study’s observational design and absence of a control group limit the ability to draw definitive conclusions about causal efficacy. Further randomized controlled trials with larger, more diverse populations and extended follow-up periods are necessary to confirm these findings and establish dupilumab as a standard treatment option in CRSwNP management.

### Study Limitations

Several limitations should be considered when interpreting the findings of this study. First, the sample size was relatively small, with only 40 patients included, which may limit the generalizability of the results to larger and more diverse populations. Additionally, this was a single-center study, and the patient demographics may not reflect those of broader populations, potentially limiting the external validity of our findings.

Second, while we monitored key clinical parameters such as symptom scores, polyp size, and biomarkers (e.g., total IgE, peripheral eosinophilia), other important markers of inflammation and disease progression, such as specific tissue biopsy analyses, were not incorporated. A more comprehensive understanding of the molecular and histopathological changes associated with treatment would have provided deeper insight into the mechanisms behind the clinical improvements observed.

Third, this study’s follow-up period was limited. While we were able to capture the short-term outcomes, a longer follow-up would be necessary to assess the sustained efficacy of dupilumab over time, as well as the potential for disease recurrence after the discontinuation of treatment.

Finally, the absence of a control group in this cohort limits our ability to directly compare the outcomes of patients receiving dupilumab with those receiving other standard treatments such as corticosteroids or surgical interventions. Future studies should include randomized controlled trials to assess the comparative effectiveness of biologic treatments in CRS more rigorously.

## Figures and Tables

**Table 1:** Baseline demographic and clinical characteristics of patients with CRSwNP.

Characteristic	Total Cohort (*n* = 40)
Age, mean (SD)	57.1 (14.3)
Gender, n (%)	
Male	22 (55%)
Female	18 (45%)
Race/ethnicity, n (%)	
Caucasian	34 (85%)
Hispanic	3 (7.5%)
Black	2 (5%)
Other	1 (2.5%)
Comorbidities, n (%)	
Asthma	16 (40%)
NSAID-exacerbated respiratory disease (NSAID-ERD)	7 (17.5%)
Inhalant allergies	16 (40%)
Baseline clinical scores, mean (SD)	
Nasal Polyp Score (NPS)	6.9 (1.0)
Sino-Nasal Outcome Test (SNOT-22)	53.6 (22.4)
Numeric Rating Scale (NRS)	7.7 (1.2)
Visual Analog Scale (VAS) for smell	72.3 (22.0)

## Data Availability

Data are contained within the article.
